# Poly[diaqua­tris(μ_4_-isophthalato)dilanthanum(III)]

**DOI:** 10.1107/S1600536809054543

**Published:** 2010-01-30

**Authors:** Le-Qing Fan, Ji-Huai Wu

**Affiliations:** a Institute of Materials Physical Chemistry and the Key Laboratory for Functional Materials of Fujian Higher Education, Huaqiao University, Quanzhou, Fujian 362021, People’s Republic of China

## Abstract

In the title coordination polymer, [La_2_(C_8_H_4_O_4_)_3_(H_2_O)_2_]_*n*_, there are two independent La^III^ atoms which are coordinated differently in slightly distorted penta­gonal-bipyramidal and slightly disorted bicapped trigonal-prismatic environments. The La^III^ ions are bridged by μ_4_-isophthalate ligands, forming two-dimensional layers. In the crystal structure, these layers are connected by inter­molecular O—H⋯O hydrogen bonds into a three-dimensional network.

## Related literature

For background information on lanthanide coordination polymers, see: Cheng *et al.* (2007 ▶); Dorweiler *et al.* (2009 ▶); Mondal *et al.* (2009 ▶) and for the use of multicarboxyl group ligands in this type of polymer, see: Mahata *et al.* (2007 ▶); Zhou *et al.* (2008 ▶).
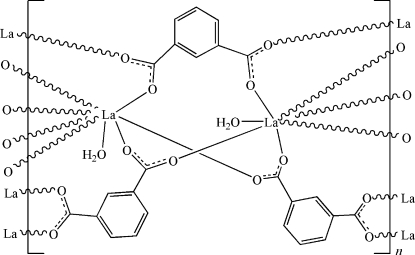


## Experimental

### 

#### Crystal data

[La_2_(C_8_H_4_O_4_)_3_(H_2_O)_2_]
*M* *_r_* = 806.19
Monoclinic, 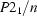

*a* = 13.3956 (12) Å
*b* = 14.4877 (8) Å
*c* = 13.5754 (11) Å
β = 103.998 (5)°
*V* = 2556.4 (3) Å^3^
*Z* = 4
Mo *K*α radiation
μ = 3.37 mm^−1^
*T* = 293 K
0.20 × 0.15 × 0.10 mm

#### Data collection

Rigaku Mercury diffractometer
Absorption correction: multi-scan (*CrystalClear*; Rigaku, 2007 ▶) *T*_min_ = 0.717, *T*_max_ = 1.000
19411 measured reflections
5841 independent reflections
5111 reflections with *I* > 2σ(*I*)
*R*_int_ = 0.042

#### Refinement

*R*[*F* ^2^ > 2σ(*F* ^2^)] = 0.048
*wR*(*F* ^2^) = 0.139
*S* = 1.08
5841 reflections
361 parameters
18 restraints
H-atom parameters constrained
Δρ_max_ = 2.72 e Å^−3^
Δρ_min_ = −1.33 e Å^−3^

### 

Data collection: *CrystalClear* (Rigaku, 2007 ▶); cell refinement: *CrystalClear*; data reduction: *CrystalClear*; program(s) used to solve structure: *SHELXS97* (Sheldrick, 2008 ▶); program(s) used to refine structure: *SHELXL97* (Sheldrick, 2008 ▶); molecular graphics: *DIAMOND* (Brandenburg, 2004 ▶); software used to prepare material for publication: *SHELXTL* (Sheldrick, 2008 ▶).

## Supplementary Material

Crystal structure: contains datablocks global, I. DOI: 10.1107/S1600536809054543/lh2972sup1.cif

Structure factors: contains datablocks I. DOI: 10.1107/S1600536809054543/lh2972Isup2.hkl

Additional supplementary materials:  crystallographic information; 3D view; checkCIF report

## Figures and Tables

**Table 1:** Hydrogen-bond geometry (Å, °)

*D*—H⋯*A*	*D*—H	H⋯*A*	*D*⋯*A*	*D*—H⋯*A*
O13—H13*A*⋯O2^i^	0.85	2.27	2.814 (8)	122
O13—H13*B*⋯O11^i^	0.85	2.20	2.976 (8)	152
O14—H14*B*⋯O10^ii^	0.85	1.97	2.795 (8)	162
O14—H14*A*⋯O7^iii^	0.85	2.10	2.656 (8)	122

Symmetry codes: (i) 
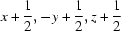
; (ii) 
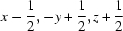
; (iii) 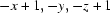.

**Fig. 1.**
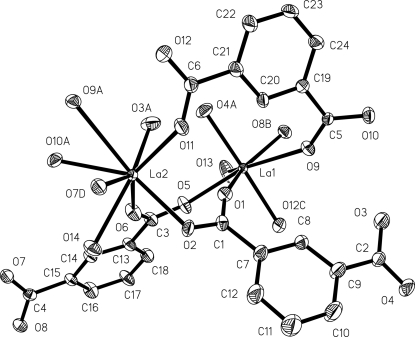
The asymmetric unit of (I), showing the atom-numbering scheme. H atoms are not included and displacement ellipsoids are drawn at the 30% probability level. Additional symmetry related atoms are included to complete the coordination geomtry around the La atoms [Symmetry codes: (A) 1.5 — x, —0.5 + y, 0.5 — z; (B) 0.5 + x, 0.5 — y, —0.5 + z; (C) 1.5 — x, 0.5 + y, 0.5 — z; (D) 1 — x, — y, 1 — z].

**Fig. 2.**
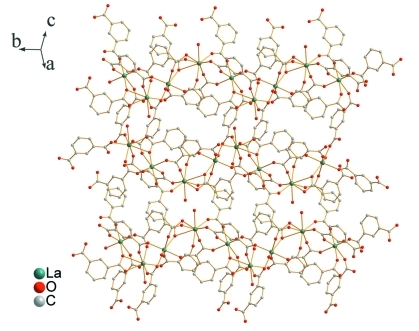
View of part of the two-dimensional layer of (I). H atoms are not included.

## supplementary crystallographic information

### Comment

Investigations of the constructions of coordination polymers based on lanthanide metals have attracted great interests not only for their diverse structures but also for their luminescent and magnetic properties (Cheng *et al.*, 2007; Dorweiler *et al.*, 2009; Mondal *et al.*, 2009). Because ligands containing multicarboxyl groups can give rise to abundant coordination modes, they have been selected to build lanthanide coordination polymers (Mahata *et al.*, 2007; Zhou *et al.*, 2008). We report herein the crystal structure of the title La(III) compound, (I).

As shown in Fig.1, the asymmetric unit of (I) contains two independent La^III^ ions, three isophthalato (ip) ligands, and two coordinated water molecules. Atom La1 is coordinated by seven O atoms in a slightly distorted pentagonal bipyramidal environment. The pentagonal plane is occupied by four oxygen atoms from four ip ligands and one oxygen atom from a water molecule, and the two apical sites are occupied by another two oxygen atoms from two ip ligands. The coordination geometry of La2 is a slightly disortoted bi-capped trigonal prism. The La^III^ ions are bridged by µ_4_-isophthalato ligands to form two-dimensional layers (Fig. 2). In the crystal structure, these layers are connected by intermolecular O—H···O hydrogen bonds to form a three-dimensional network.

### Experimental

A mixture of La_2_O_3_ (0.163 g, 0.5 mmol), isophthalic acid (0.166 g, 1 mmol) and H_2_O (10 ml) was placed in a 23 ml Teflon-lined reactor, which was heated to 443 K for 7 days and then cooled to room temperature at a rate of 0.2 K h^—1^. The colorless crystals obtained were washed with water and dried in air (yield 16% based on La).

### Refinement

H atoms were positioned geometrically and refined as riding atoms, with C—H = 0.93 Å, O—H = 0.85 Å, *U*_iso_(H) = 1.2*U*_eq_(C or O).

### Crystal data

**Table:**

[La_2_(C_8_H_4_O_4_)_3_(H_2_O)_2_]	*F*(000) = 1544
*M**_r_* = 806.19	*D*_x_ = 2.095 Mg m^−^^3^
Monoclinic, *P*2_1_/*n*	Mo *K*α radiation, λ = 0.71073 Å
Hall symbol: -P 2yn	Cell parameters from 6025 reflections
*a* = 13.3956 (12) Å	θ = 3.1–27.5°
*b* = 14.4877 (8) Å	µ = 3.37 mm^−^^1^
*c* = 13.5754 (11) Å	*T* = 293 K
β = 103.998 (5)°	Prism, colorless
*V* = 2556.4 (3) Å^3^	0.20 × 0.15 × 0.10 mm
*Z* = 4	

### Data collection

**Table:**

Rigaku Mercury diffractometer	5841 independent reflections
Radiation source: fine-focus sealed tube	5111 reflections with *I* > 2σ(*I*)
graphite	*R*_int_ = 0.042
ω scans	θ_max_ = 27.5°, θ_min_ = 2.1°
Absorption correction: multi-scan (*CrystalClear*; Rigaku, 2007)	*h* = −17→16
*T*_min_ = 0.717, *T*_max_ = 1.000	*k* = −18→18
19411 measured reflections	*l* = −17→17

### Refinement

**Table:**

Refinement on *F*^2^	Primary atom site location: structure-invariant direct methods
Least-squares matrix: full	Secondary atom site location: difference Fourier map
*R*[*F*^2^ > 2σ(*F*^2^)] = 0.048	Hydrogen site location: inferred from neighbouring sites
*wR*(*F*^2^) = 0.139	H-atom parameters constrained
*S* = 1.08	*w* = 1/[σ^2^(*F*_o_^2^) + (0.0811*P*)^2^ + 4.3612*P*] where *P* = (*F*_o_^2^ + 2*F*_c_^2^)/3
5841 reflections	(Δ/σ)_max_ = 0.001
361 parameters	Δρ_max_ = 2.72 e Å^−^^3^
18 restraints	Δρ_min_ = −1.33 e Å^−^^3^

### Special details

**Table:**

Geometry. All esds (except the esd in the dihedral angle between two l.s. planes) are estimated using the full covariance matrix. The cell esds are taken into account individually in the estimation of esds in distances, angles and torsion angles; correlations between esds in cell parameters are only used when they are defined by crystal symmetry. An approximate (isotropic) treatment of cell esds is used for estimating esds involving l.s. planes.
Refinement. Refinement of *F*^2^ against ALL reflections. The weighted *R*-factor wR and goodness of fit *S* are based on *F*^2^, conventional *R*-factors *R* are based on *F*, with *F* set to zero for negative *F*^2^. The threshold expression of *F*^2^ > σ(*F*^2^) is used only for calculating *R*-factors(gt) etc. and is not relevant to the choice of reflections for refinement. *R*-factors based on *F*^2^ are statistically about twice as large as those based on *F*, and *R*- factors based on ALL data will be even larger.

### Fractional atomic coordinates and isotropic or equivalent isotropic displacement parameters (Å^2^)

**Table:**

	*x*	*y*	*z*	*U*_iso_*/*U*_eq_	
La1	0.84385 (2)	0.31714 (2)	0.38814 (2)	0.02027 (12)	
La2	0.61501 (2)	0.06678 (2)	0.32769 (2)	0.02238 (12)	
O1	0.6774 (3)	0.2657 (3)	0.2958 (3)	0.0358 (10)	
O2	0.5239 (4)	0.2049 (3)	0.2615 (4)	0.0375 (11)	
O3	0.7107 (4)	0.5678 (4)	0.1326 (5)	0.0545 (15)	
O4	0.5900 (4)	0.6718 (3)	0.1260 (5)	0.0510 (14)	
O5	0.7672 (4)	0.2617 (4)	0.5144 (4)	0.0463 (13)	
O6	0.6532 (4)	0.1469 (4)	0.4850 (4)	0.0463 (13)	
O7	0.5312 (4)	0.0136 (3)	0.7733 (4)	0.0383 (11)	
O8	0.5033 (4)	0.1180 (3)	0.8868 (3)	0.0392 (11)	
O9	0.8372 (4)	0.3771 (3)	0.2105 (3)	0.0359 (10)	
O10	0.8799 (4)	0.4248 (3)	0.0703 (4)	0.0395 (11)	
O11	0.6533 (4)	0.0748 (3)	0.1637 (4)	0.0437 (12)	
O12	0.7235 (4)	−0.0402 (3)	0.0943 (4)	0.0482 (13)	
O13	0.9519 (5)	0.3449 (5)	0.5562 (4)	0.071 (2)	
H13B	1.0027	0.3819	0.5703	0.085*	
H13A	0.9312	0.3119	0.5991	0.085*	
O14	0.4569 (4)	0.0722 (3)	0.3964 (4)	0.0454 (13)	
H14A	0.4165	0.0455	0.3466	0.054*	
H14B	0.4465	0.0688	0.4557	0.054*	
C1	0.5822 (5)	0.2719 (5)	0.2627 (5)	0.0319 (13)	
C2	0.6223 (5)	0.5905 (5)	0.1368 (5)	0.0381 (15)	
C3	0.7040 (5)	0.2086 (5)	0.5416 (5)	0.0373 (15)	
C4	0.5455 (5)	0.0912 (5)	0.8170 (5)	0.0318 (13)	
C5	0.8589 (4)	0.3606 (4)	0.1257 (4)	0.0265 (12)	
C6	0.7158 (6)	0.0436 (5)	0.1157 (5)	0.0366 (15)	
C7	0.5369 (5)	0.3625 (5)	0.2226 (6)	0.0409 (16)	
C8	0.5967 (5)	0.4312 (4)	0.1954 (6)	0.0365 (15)	
H8A	0.6664	0.4201	0.2018	0.044*	
C9	0.5563 (6)	0.5166 (5)	0.1589 (7)	0.0497 (19)	
C10	0.4527 (8)	0.5310 (7)	0.1490 (10)	0.082 (3)	
H10A	0.4231	0.5864	0.1219	0.099*	
C11	0.3917 (9)	0.4641 (9)	0.1789 (11)	0.098 (4)	
H11A	0.3229	0.4759	0.1765	0.118*	
C12	0.4353 (7)	0.3788 (6)	0.2126 (9)	0.072 (3)	
H12A	0.3939	0.3324	0.2285	0.086*	
C13	0.6907 (5)	0.2169 (5)	0.6486 (5)	0.0353 (14)	
C14	0.6329 (5)	0.1517 (5)	0.6845 (5)	0.0339 (14)	
H14C	0.6048	0.1024	0.6431	0.041*	
C15	0.6156 (5)	0.1586 (5)	0.7833 (5)	0.0320 (13)	
C16	0.6589 (6)	0.2315 (6)	0.8462 (5)	0.0457 (18)	
H16A	0.6474	0.2371	0.9108	0.055*	
C17	0.7189 (6)	0.2949 (6)	0.8120 (6)	0.0466 (19)	
H17A	0.7495	0.3426	0.8546	0.056*	
C18	0.7350 (6)	0.2888 (5)	0.7121 (6)	0.0428 (16)	
H18A	0.7751	0.3327	0.6896	0.051*	
C19	0.8584 (5)	0.2627 (4)	0.0888 (5)	0.0320 (13)	
C20	0.7905 (5)	0.2005 (4)	0.1165 (5)	0.0337 (14)	
H20A	0.7471	0.2201	0.1564	0.040*	
C21	0.7878 (5)	0.1090 (5)	0.0845 (6)	0.0399 (16)	
C22	0.8536 (7)	0.0792 (5)	0.0250 (7)	0.056 (2)	
H22A	0.8520	0.0182	0.0033	0.067*	
C23	0.9201 (7)	0.1409 (5)	−0.0010 (7)	0.059 (2)	
H23A	0.9649	0.1210	−0.0393	0.071*	
C24	0.9221 (6)	0.2322 (5)	0.0284 (6)	0.0427 (17)	
H24A	0.9662	0.2734	0.0078	0.051*	

### Atomic displacement parameters (Å^2^)

**Table:**

	*U*^11^	*U*^22^	*U*^33^	*U*^12^	*U*^13^	*U*^23^
La1	0.02440 (19)	0.01671 (19)	0.02177 (19)	0.00019 (11)	0.00959 (13)	0.00025 (11)
La2	0.0265 (2)	0.0186 (2)	0.02394 (19)	−0.00009 (12)	0.00989 (14)	0.00050 (11)
O1	0.033 (2)	0.039 (3)	0.034 (2)	0.000 (2)	0.0058 (19)	0.001 (2)
O2	0.039 (3)	0.024 (2)	0.049 (3)	−0.003 (2)	0.009 (2)	0.008 (2)
O3	0.035 (3)	0.046 (3)	0.083 (4)	−0.002 (2)	0.015 (3)	0.017 (3)
O4	0.047 (3)	0.027 (3)	0.079 (4)	−0.002 (2)	0.016 (3)	0.008 (2)
O5	0.051 (3)	0.058 (3)	0.035 (3)	−0.018 (3)	0.021 (2)	0.001 (2)
O6	0.047 (3)	0.058 (3)	0.036 (3)	−0.018 (3)	0.014 (2)	−0.009 (2)
O7	0.038 (3)	0.039 (3)	0.041 (3)	−0.005 (2)	0.015 (2)	−0.002 (2)
O8	0.041 (3)	0.048 (3)	0.031 (2)	−0.001 (2)	0.0141 (19)	−0.003 (2)
O9	0.040 (2)	0.039 (3)	0.031 (2)	−0.007 (2)	0.0132 (19)	−0.0029 (19)
O10	0.056 (3)	0.029 (2)	0.036 (3)	−0.008 (2)	0.017 (2)	0.0010 (19)
O11	0.057 (3)	0.040 (3)	0.043 (3)	−0.011 (2)	0.027 (2)	−0.003 (2)
O12	0.060 (3)	0.030 (3)	0.061 (3)	−0.002 (2)	0.027 (3)	−0.003 (2)
O13	0.068 (4)	0.105 (5)	0.035 (3)	−0.049 (4)	0.006 (3)	0.004 (3)
O14	0.046 (3)	0.058 (3)	0.039 (3)	−0.007 (2)	0.024 (2)	−0.013 (2)
C1	0.038 (3)	0.031 (3)	0.027 (3)	−0.005 (3)	0.008 (2)	0.001 (2)
C2	0.034 (4)	0.032 (4)	0.047 (4)	0.001 (3)	0.008 (3)	0.008 (3)
C3	0.035 (3)	0.043 (4)	0.036 (3)	−0.007 (3)	0.013 (3)	−0.004 (3)
C4	0.025 (3)	0.039 (4)	0.030 (3)	0.002 (3)	0.004 (2)	0.000 (3)
C5	0.025 (3)	0.024 (3)	0.031 (3)	0.000 (2)	0.008 (2)	0.004 (2)
C6	0.049 (4)	0.031 (3)	0.034 (3)	−0.003 (3)	0.016 (3)	0.000 (3)
C7	0.033 (3)	0.029 (4)	0.057 (4)	−0.004 (3)	0.005 (3)	0.006 (3)
C8	0.032 (3)	0.033 (4)	0.046 (4)	−0.001 (3)	0.012 (3)	0.005 (3)
C9	0.034 (4)	0.035 (4)	0.077 (6)	0.003 (3)	0.009 (4)	0.011 (4)
C10	0.055 (5)	0.046 (5)	0.149 (8)	0.009 (4)	0.029 (5)	0.036 (5)
C11	0.060 (6)	0.079 (6)	0.160 (9)	0.010 (5)	0.034 (6)	0.044 (6)
C12	0.051 (5)	0.041 (4)	0.127 (7)	−0.003 (4)	0.029 (5)	0.023 (5)
C13	0.037 (3)	0.041 (4)	0.032 (3)	−0.007 (3)	0.017 (3)	−0.006 (3)
C14	0.033 (3)	0.040 (4)	0.032 (3)	−0.004 (3)	0.012 (2)	−0.003 (3)
C15	0.031 (3)	0.039 (4)	0.028 (3)	−0.002 (3)	0.011 (2)	−0.001 (3)
C16	0.052 (4)	0.057 (5)	0.029 (3)	−0.017 (4)	0.012 (3)	−0.011 (3)
C17	0.046 (4)	0.055 (5)	0.045 (4)	−0.026 (4)	0.023 (3)	−0.022 (4)
C18	0.046 (4)	0.042 (4)	0.045 (4)	−0.008 (3)	0.019 (3)	−0.004 (3)
C19	0.036 (3)	0.027 (3)	0.034 (3)	0.000 (3)	0.011 (3)	−0.001 (3)
C20	0.044 (4)	0.026 (3)	0.034 (3)	−0.006 (3)	0.017 (3)	0.002 (3)
C21	0.043 (4)	0.033 (4)	0.050 (4)	−0.008 (3)	0.022 (3)	−0.004 (3)
C22	0.073 (6)	0.032 (4)	0.078 (6)	−0.013 (4)	0.049 (5)	−0.005 (4)
C23	0.081 (6)	0.034 (4)	0.083 (6)	−0.006 (4)	0.061 (5)	−0.012 (4)
C24	0.044 (4)	0.036 (4)	0.055 (4)	−0.004 (3)	0.027 (3)	−0.002 (3)

### Geometric parameters (Å, °)

**Table:**

La1—O12^i^	2.291 (5)	C1—C7	1.492 (9)
La1—O4^ii^	2.310 (5)	C2—C9	1.465 (10)
La1—O8^iii^	2.338 (5)	C3—C13	1.510 (9)
La1—O5	2.343 (5)	C4—C15	1.500 (9)
La1—O1	2.396 (4)	C5—C19	1.504 (9)
La1—O13	2.422 (5)	C5—La2^i^	3.056 (6)
La1—O9	2.545 (4)	C6—C21	1.486 (10)
La2—O3^ii^	2.266 (5)	C7—C12	1.356 (11)
La2—O6	2.376 (5)	C7—C8	1.383 (9)
La2—O2	2.402 (4)	C8—C9	1.392 (10)
La2—O7^iv^	2.403 (5)	C8—H8A	0.9300
La2—O11	2.405 (5)	C9—C10	1.377 (12)
La2—O10^ii^	2.471 (5)	C10—C11	1.391 (14)
La2—O14	2.514 (5)	C10—H10A	0.9300
La2—O9^ii^	2.897 (5)	C11—C12	1.395 (14)
O1—C1	1.250 (8)	C11—H11A	0.9300
O2—C1	1.243 (8)	C12—H12A	0.9300
O3—C2	1.244 (9)	C13—C14	1.382 (9)
O3—La2^i^	2.266 (5)	C13—C18	1.391 (10)
O4—C2	1.251 (8)	C14—C15	1.418 (9)
O4—La1^i^	2.310 (5)	C14—H14C	0.9300
O5—C3	1.263 (8)	C15—C16	1.394 (10)
O6—C3	1.264 (8)	C16—C17	1.373 (10)
O7—C4	1.265 (8)	C16—H16A	0.9300
O7—La2^iv^	2.403 (5)	C17—C18	1.426 (10)
O8—C4	1.276 (8)	C17—H17A	0.9300
O8—La1^v^	2.338 (5)	C18—H18A	0.9300
O9—C5	1.276 (7)	C19—C24	1.391 (9)
O9—La2^i^	2.897 (5)	C19—C20	1.395 (9)
O10—C5	1.269 (7)	C20—C21	1.393 (9)
O10—La2^i^	2.471 (5)	C20—H20A	0.9300
O11—C6	1.262 (8)	C21—C22	1.400 (10)
O12—C6	1.258 (8)	C22—C23	1.367 (10)
O12—La1^ii^	2.291 (5)	C22—H22A	0.9300
O13—H13B	0.8500	C23—C24	1.379 (11)
O13—H13A	0.8501	C23—H23A	0.9300
O14—H14A	0.8500	C24—H24A	0.9300
O14—H14B	0.8501		
			
O12^i^—La1—O4^ii^	178.4 (2)	C7—C1—La2	164.5 (5)
O12^i^—La1—O8^iii^	91.26 (18)	O3—C2—O4	123.4 (7)
O4^ii^—La1—O8^iii^	89.77 (18)	O3—C2—C9	116.3 (6)
O12^i^—La1—O5	88.85 (19)	O4—C2—C9	120.3 (7)
O4^ii^—La1—O5	89.6 (2)	O5—C3—O6	123.5 (6)
O8^iii^—La1—O5	134.94 (17)	O5—C3—C13	118.4 (6)
O12^i^—La1—O1	89.71 (19)	O6—C3—C13	118.0 (6)
O4^ii^—La1—O1	90.01 (18)	O7—C4—O8	125.1 (6)
O8^iii^—La1—O1	148.99 (15)	O7—C4—C15	118.1 (6)
O5—La1—O1	76.07 (17)	O8—C4—C15	116.8 (6)
O12^i^—La1—O13	85.0 (2)	O10—C5—O9	121.9 (6)
O4^ii^—La1—O13	94.3 (2)	O10—C5—C19	118.6 (5)
O8^iii^—La1—O13	66.51 (17)	O9—C5—C19	119.5 (5)
O5—La1—O13	68.62 (19)	O10—C5—La2^i^	51.4 (3)
O1—La1—O13	144.36 (18)	O9—C5—La2^i^	70.7 (3)
O12^i^—La1—O9	82.32 (17)	C19—C5—La2^i^	169.2 (4)
O4^ii^—La1—O9	99.13 (19)	O12—C6—O11	124.5 (6)
O8^iii^—La1—O9	71.23 (15)	O12—C6—C21	117.0 (6)
O5—La1—O9	152.80 (17)	O11—C6—C21	118.5 (6)
O1—La1—O9	78.20 (15)	C12—C7—C8	118.7 (7)
O13—La1—O9	135.42 (17)	C12—C7—C1	120.0 (7)
O3^ii^—La2—O6	78.3 (2)	C8—C7—C1	121.4 (6)
O3^ii^—La2—O2	119.00 (18)	C7—C8—C9	122.3 (7)
O6—La2—O2	84.73 (19)	C7—C8—H8A	118.9
O3^ii^—La2—O7^iv^	142.19 (18)	C9—C8—H8A	118.9
O6—La2—O7^iv^	135.92 (17)	C10—C9—C8	117.8 (7)
O2—La2—O7^iv^	85.41 (16)	C10—C9—C2	120.8 (7)
O3^ii^—La2—O11	77.4 (2)	C8—C9—C2	121.4 (7)
O6—La2—O11	139.53 (17)	C9—C10—C11	121.0 (9)
O2—La2—O11	79.43 (17)	C9—C10—H10A	119.5
O7^iv^—La2—O11	79.77 (17)	C11—C10—H10A	119.5
O3^ii^—La2—O10^ii^	89.1 (2)	C10—C11—C12	119.0 (10)
O6—La2—O10^ii^	86.12 (17)	C10—C11—H11A	120.5
O2—La2—O10^ii^	147.73 (16)	C12—C11—H11A	120.5
O7^iv^—La2—O10^ii^	79.90 (17)	C7—C12—C11	121.2 (9)
O11—La2—O10^ii^	125.15 (16)	C7—C12—H12A	119.4
O3^ii^—La2—O14	145.5 (2)	C11—C12—H12A	119.4
O6—La2—O14	70.65 (17)	C14—C13—C18	119.0 (6)
O2—La2—O14	73.46 (17)	C14—C13—C3	119.4 (6)
O7^iv^—La2—O14	65.34 (16)	C18—C13—C3	121.6 (6)
O11—La2—O14	136.85 (18)	C13—C14—C15	121.3 (6)
O10^ii^—La2—O14	74.30 (17)	C13—C14—H14C	119.4
O3^ii^—La2—O9^ii^	77.81 (16)	C15—C14—H14C	119.4
O6—La2—O9^ii^	128.00 (17)	C16—C15—C14	119.6 (6)
O2—La2—O9^ii^	146.91 (15)	C16—C15—C4	120.3 (6)
O7^iv^—La2—O9^ii^	67.88 (15)	C14—C15—C4	120.0 (6)
O11—La2—O9^ii^	76.96 (15)	C17—C16—C15	119.3 (7)
O10^ii^—La2—O9^ii^	48.19 (14)	C17—C16—H16A	120.3
O14—La2—O9^ii^	109.79 (15)	C15—C16—H16A	120.3
C1—O1—La1	154.9 (4)	C16—C17—C18	121.1 (7)
C1—O2—La2	112.7 (4)	C16—C17—H17A	119.5
C2—O3—La2^i^	158.2 (6)	C18—C17—H17A	119.5
C2—O4—La1^i^	137.7 (5)	C13—C18—C17	119.7 (7)
C3—O5—La1	150.0 (5)	C13—C18—H18A	120.1
C3—O6—La2	149.2 (5)	C17—C18—H18A	120.1
C4—O7—La2^iv^	135.1 (4)	C24—C19—C20	119.2 (6)
C4—O8—La1^v^	134.3 (4)	C24—C19—C5	122.8 (6)
C5—O9—La1	145.9 (4)	C20—C19—C5	118.1 (6)
C5—O9—La2^i^	84.7 (3)	C21—C20—C19	120.0 (6)
La1—O9—La2^i^	122.32 (17)	C21—C20—H20A	120.0
C5—O10—La2^i^	105.0 (4)	C19—C20—H20A	120.0
C6—O11—La2	141.0 (5)	C20—C21—C22	120.1 (6)
C6—O12—La1^ii^	142.2 (5)	C20—C21—C6	119.2 (6)
La1—O13—H13B	125.4	C22—C21—C6	120.7 (6)
La1—O13—H13A	109.3	C23—C22—C21	119.1 (7)
H13B—O13—H13A	125.2	C23—C22—H22A	120.4
La2—O14—H14A	96.9	C21—C22—H22A	120.4
La2—O14—H14B	134.0	C22—C23—C24	121.4 (7)
H14A—O14—H14B	120.6	C22—C23—H23A	119.3
O2—C1—O1	122.1 (6)	C24—C23—H23A	119.3
O2—C1—C7	118.9 (6)	C23—C24—C19	120.2 (7)
O1—C1—C7	119.0 (6)	C23—C24—H24A	119.9
O2—C1—La2	45.6 (3)	C19—C24—H24A	119.9
O1—C1—La2	76.5 (4)		

Symmetry codes: (i) −*x*+3/2, *y*+1/2, −*z*+1/2; (ii) −*x*+3/2, *y*−1/2, −*z*+1/2; (iii) *x*+1/2, −*y*+1/2, *z*−1/2; (iv) −*x*+1, −*y*, −*z*+1; (v) *x*−1/2, −*y*+1/2, *z*+1/2.

### Hydrogen-bond geometry (Å, °)

**Table:**

*D*—H···*A*	*D*—H	H···*A*	*D*···*A*	*D*—H···*A*
O13—H13A···O2^vi^	0.85	2.27	2.814 (8)	122
O13—H13B···O11^vi^	0.85	2.20	2.976 (8)	152
O14—H14B···O10^v^	0.85	1.97	2.795 (8)	162
O14—H14A···O7^iv^	0.85	2.10	2.656 (8)	122

Symmetry codes: (vi) *x*+1/2, −*y*+1/2, *z*+1/2; (v) *x*−1/2, −*y*+1/2, *z*+1/2; (iv) −*x*+1, −*y*, −*z*+1.
